# Increasing community capacity to address local environmental health concerns using a community grant program in Atlanta, Georgia

**DOI:** 10.21203/rs.3.rs-7724569/v1

**Published:** 2026-01-14

**Authors:** Erin Lebow-Skelley, Dana H.Z. Williamson, Laura Whitaker, Simone Charles, Camilla Warren, Lynne Young, Michelle C. Kegler, Melanie A. Pearson

**Affiliations:** Emory University; HERCULES Stakeholder Advisory Board; RAND Corporation; University of Michigan–Ann Arbor; HERCULES Stakeholder Advisory Board; Pathways to Sustainability; Emory University; Emory University

**Keywords:** Community capacity, community grants, environmental health, community engagement, evaluation

## Abstract

**Background:**

Communities play a central role in responding to environmental exposures that negatively impact them, and growing their capacity to respond to these environmental threats is one approach to protect and improve community health. The Emory University HERCULES Exposome Research Center and its Stakeholder Advisory Board developed a community grant program with the goal of increasing the capacity of communities in Atlanta, Georgia to address their local environmental health concerns. This paper presents the community capacity outcomes of that program, demonstrating how a small grant program has the potential to enhance communities’ capacity to address environmental health concerns.

**Methods:**

To evaluate the community capacity outcomes of the Community Grant Program we conducted in-depth semi-structured interviews with organizational representatives after they completed the program. The interview guide assessed dimensions of community capacity: leadership, knowledge and skills, networks and partnerships, resources, sense of community, and community power. Two team members independently transcribed and coded the, resolving discrepancies through discussion. We used NVivo qualitative analysis software to manage and analyze the data, and conducted secondary analysis of code reports to identify the themes reported here.

**Results:**

We interviewed a representative from each of the 12 grantee organizations. Grantees showed increased capacity with regards to leadership, sense of community, community power, knowledge, skills, and expanded networks and partnerships. For example, the program increased opportunities to take on leadership roles, to build community trust, to increase community awareness of environmental health issues, and for community members to act.

**Conclusions:**

Community grant programs may be a novel way to support and enhance community capacity. Using community capacity frameworks to guide community grant programming can produce short-term outcomes while building sustained capacity for long-term goals. This program not only provided financial support but also served as a catalyst for fostering leadership, knowledge, and skills, enhancing a sense of community, empowering individuals and communities to act, and expanding networks and partnerships. Overall, the success of this small Community Grant Program underscores the importance of investing in grassroots initiatives and collaborative efforts to enhance community capacity to address complex social and environmental challenges.

## Background

### Community Capacity for Environmental Health

Central to the field of environmental health is the relationship between people and their environment and efforts to reduce harmful exposures where people live, learn, work and play (1). Communities, especially those defined geographically, play a central role in responding to those exposures, and community capacity is a framework that helps explore how communities respond and how to improve that response (2). Community capacity has been defined by Goodman et al. (3) as the “the characteristics of communities that affect their ability to identify, mobilize, and address social and public health problems.” (p259). Strengthening community capacity can increase the ability of a community to protect itself and improve environmental health (2). Community capacity building includes strengthening a community’s ability to enhance decision making, collective action, and become self-reliant by increasing social cohesion (4) and social capital (5–9). In this paper, we explore the extent to which community capacity was built through a community grant program.

### The HERCULES Exposome Research Center

The HERCULES Exposome Research Center (HERCULES) is an environmental health research center at Emory University funded by the National Institute of Environmental Health Sciences to advance exposome research (P30ES019776) (10, 11). The Exposome is the concept that environmental exposures play a role in our health over a lifetime (12). These exposures include what we eat and drink, the air we breathe, our behaviors and lifestyles, and where we live, work, and play.

The HERCULES Community Engagement Core (CEC) aims to create multi-directional dialogue between Atlanta communities and the Center, to increase the capacity of faculty and students to conduct community-engaged research, and to increase the capacity of local communities to address their exposome and environmental health concerns. The HERCULES Stakeholder Advisory Board (SAB) plays a crucial role in establishing communication between the Center and environmental health stakeholders in the Atlanta-metro area, and includes representatives from local communities, community-based and national nonprofits, academia, and local, state, and federal government agencies. The SAB was a guiding force for ensuring that funding and expertise from the Center went to local Atlanta-area communities to increase their capacity to address their environmental health concerns. Most residents in the Atlanta area are people of color (13) who face myriad environmental health threats such as a combined water/sewer system that contributes to excess flooding and sewer overflows (14–17) and pollution sources that are predominantly located in areas with a high population of color (18). Members of the SAB, along with others in the field of community engagement, recognize that these same communities are the best situated to take action and implement solutions in their own communities and that HERCULES could support their capacity to do so (19, 20). Together with the CEC, the SAB identified community grants as a mechanism to do this and co-developed the HERCULES Clarence “Shaheed” DuBois Community Grant Program (Community Grant Program).

### Community grants, partnerships, and community capacity

The Community Grant Program provided a limited amount of funding ($2500 over 12 months, determined as adequate by the SAB) to community-based organizations in the metro-Atlanta area to implement a project or program that addressed local exposome-related concerns. While each grantee had project-specific goals and outcomes ranging from healthy food access to addressing illegal dumping and water pollution, the goal of the program was also to enhance their capacity in order to sustain their efforts beyond the one-year funding period and address new challenges as they arise.

Community grants, or mini-grants, are a well-established model for forming partnerships between research institutions and communities, with the potential to provide tangible results to the communities, increase community capacity, and improve and inform research (21–24). For example, Kegler et al. (23) describe three mini-grant models used to seed community-academic research partnerships, one of which specifically aimed to increase collaborative research capacity. The Centers for Disease Control and Prevention’s Cancer Prevention and Control Research Network has used mini-grants to build community capacity for implementing evidence-based health promotion strategies (21, 24), and Deacon and colleagues (22) found that their mini-grant program mobilized residents and built readiness and capacity for change. Building from these established models, the HERCULES CEC and SAB developed the Community Grant Program to build partnerships with additional communities in metro-Atlanta while also enhancing their capacity to address their exposome-related concerns. Pearson et al. (25) describe the program implementation process and outcomes related to accomplishments, sustainability, and partnerships. Briefly, from 2014–2016 a SAB review committee selected 12 distinct non-profit organizations to receive 13 Community Grants (one organization received two grants). Aligned with our community engagement goals, the Grant Program successfully built relationships between HERCULES and local organizations in the Atlanta area. Furthermore, the grantees identified accomplishments such as increased awareness and credibility of their organization, increased knowledge among residents, and plans for sustainability that included acquiring additional funding and institutionalizing their programming. We present the community capacity outcomes here, demonstrating how a small community grant program has the potential to enhance communities’ capacity to address environmental health concerns.

## Methods

We modeled the Community Grant Program evaluation plan after the program evaluation for another capacity-building program co-led by co-author Kegler (The Tobacco Technical Assistance Consortium (TTAC)) (26) who also contributed to the foundational community capacity reference “Identifying and Defining the Dimensions of Community Capacity to Provide a Basis for Measurement” (3). In particular, we adapted the TTAC evaluation’s in-depth semi-structured interview guide for our evaluation. This guide assessed the dimensions of community capacity as defined by Goodman et al. (3), a commonly used framework for assessing capacity building interventions (27) and included questions that were relevant to leadership, knowledge and skills, networks and partnerships, resources, sense of community, and community power. Table 1 presents the dimensions of community capacity as defined by Goodman et al. (3), and how these dimensions were operationalized for our interview guide and codebook. See Additional File 1 for the full Interview Guide.

A trained HERCULES staff member who was not involved in the grant program conducted 30-minute interviews with a representative from each of the 12 grantee organizations within two months of completion of the 12-month grant program. The interviews were audio-recorded and transcribed verbatim. The evaluation team used the interview guide to create an initial codebook, which was refined after reviewing one transcript and revising collaboratively. The codebook allowed for double-coding, acknowledging the conceptual linkages between the dimensions of community capacity (3). Two team members independently coded each transcript and met to discuss and resolve discrepancies. NVivo qualitative analysis software was used to manage and analyze the interview data, and to produce code reports once coding discrepancies were resolved (QSR International Pty Ltd. Version 11, 2015). Using the code reports, a second round of coding was conducted to identify themes and subthemes within each code, that were then organized into matrices for further review to ensure trustworthiness of the results (28).

The themes relevant to community capacity are reported as follows and organized by the dimensions of community capacity.

## Results

A representative from each of the 12 grantee organizations participated in the interviews. Grantees represented organizations across two metro-Atlanta counties, with most working in South or Southwest Atlanta. At the time the grant was received, most of the grantee organizations had existed for 10 or fewer years (n = 9), had five or fewer staff (n = 7), and had five or fewer existing partnerships (n = 9). Of the 10 grantees who reported it, most had an annual budget below $20,000 (n = 6), with most of those operating with less than $10,000. Few grantees had more than 11 existing partnerships (n = 2) and staff (n = 1), had existed for more than 10 years (n = 3), and had an annual budget over $100,000 (n = 3) (See Table 2). Additional grantee details and results are reported elsewhere (25). Here, we summarize how the Community Grant Program influenced six dimensions of community capacity outlined in Table 1.

### Leadership Development

Grantees described the extent to which the grant program provided the grantee and/or community members leadership opportunities, which included speaking publicly about their organization or empowering community members to lead aspects of the project. Grantees said the grant provided leadership opportunities for both themselves and participating community members. Community members had public speaking opportunities, gave presentations, took on leadership roles within the project, and participated in government or professional meetings about their efforts and environmental concern. For example, this grantee described the public speaking opportunity the grant provided for youth:

“… [The youth] presented those to the college students at [the local university] on Saturdays when they came in to get their breakfast or lunch, and they would stop and say “do you know about [The] Creek, well I do let me show you my diorama and let me educate you about [The] Creek”. And so it was really exceptional.” - Regarding youth aged 9–12

Grantees felt they increased their own visibility or role as leaders in their field and shared ways in which the grant provided them professional development opportunities and increased their confidence as leaders. For example, this grantee described how the structure of the grant program gave them professional development experience and grew their public speaking confidence:

I think the requirements of HERCULES in terms of like being specific about changes, being very measured, and then communicating that well, I think that’s something that I do, but I think sort of the confines that HERCULES put it in were really helpful just for practicing being very specific, but also then communicating that, and I -- Personally, I was pretty intimidated to present at the end of the year meeting, but I got a lot out of it, and I really enjoyed it in the end.

The Program also increased the confidence of some community members, such as this grantee who described the women who participated in their project:

…the women gained a lot. Some of them were very shy, and they ended up developing that confidence of being able to speak in public, like going to knock your neighbors’ doors… They were able to establish relationships with knocking on the doors and saying this is who I am, this is what I’m working on.

### Sense of Community & Community Engagement

We assessed whether the grant program fostered a sense of community, which included having an emotional connection with the community, engagement in and awareness of community issues, service to others, and community involvement. Related to the key definition facets by Goodman et al. (3) our survey revealed five main themes: trust, awareness and concern for community issues, community-engaged programming, and the importance of youth as community members. Additionally, a smaller subset of grantees expressed their sense of connection with the community in which they implemented their project.

Regarding trust and an increased sense of respect in, and connection with, the community, grantees shared that the funding allowed them to increase their organization’s visibility within the community, by either initiating new relationships or enhancing existing ones.

Of course, you know, we have a long way to go, but your grant -- I call it seed. You know, it was a spark for us to bring people together and to do more outreach and some of those other things that you have, but it was more of a, I would say outreach and relationship building, and so people were able to come and get engaged and see you know, for their selves…

Grantees also shared how the grant increased engagement in, and awareness of, environmental issues in the community. Some stated that their own involvement in the issues was enhanced by being physically present in the community, which led to programming that was driven by the community.

…not only did we do classroom settings of the education to the women, but at the same time, doing a follow up when they did the home visit to also knowing the various communities that they wanted to have the clean-up exercise. That was more to me because some of those apartment complexes I haven’t been before, but because of the project, us going there, I had to personally be involved and see what they were talking about, … and to personally be present with the cleanup.

### Community Power

We defined community power as increasing community organization or action around issues, and grantees described how their projects increased community power by providing both education and opportunities for action. Several grantees said their projects empowered community members to act by creating opportunities for them to get involved and take on leadership roles in the project (and sustain it after the project period ended). In this way, community power to act and leadership were interrelated.

And so what we’re about to do now is try to pull all the growers together in the community and look at those people who are really interested in growing food to help sustain some of these growing food projects. You know, it’s always great when you can bring things into a community to help them, but when you’re able to grow it from inside of that community and get buy in literally from the ground up, you have greater success with sustaining the project, so really feel good about where we are and where we’ve been, what we’ve started.

### Two of our grantees also specifically spoke about empowering the youth in their community, for example:

…Just going out there and participating in activities and just being on the park, and then, you know, one person -- You see one person, then the other people come… and then as far as yes, some of the young people, you know, without anybody telling them, I saw a garbage hanging here, a garbage bag hanging there, so they did that. So that is leadership. So you know, if you just get a small change, that’s a change, you know?

### Knowledge and Skills

All but one grantee reported a gain in knowledge and skills for either themselves or their community members as a result of participating in the Community Grant Program. Most grantees reported that they themselves gained some kind of knowledge or skills; and most of these specifically gained skills in program implementation and grant administration such as tracking and reporting their progress, evaluation, and budgeting. For example, this grantee shared how the grant gave them additional experience in program implementation:

…learning is an experience because like when you’re driving a car the first time, you’re scared, first time learner, the more you practice, the more you know, the more you are involved with implementation, designing, evaluation, then you master the program.

### And this grantee shared how the grant helped them improve their administrative processes:

And then I think it sort of administratively, I think, the grant process was good in terms of helping us maybe always be thoughtful about what we’re doing and keeping records, and kind of having specific metrics of this is what we consider success to be on the farm, or in terms of like the health of the plants, and the health of the soil, and the health of the water. So, I think for us it was the grant process made us be very thoughtful, and probably more specific than we’ve been in the past about what we’re trying to measure, and yes, and our success.

Half the grantees also said that their community members gained knowledge or skills as a result of the grant. About half also reported a gain in knowledge about the environment and health, either for themselves or their participants. This included knowledge around soil health, the watershed, and secondhand smoke, for example:

It’s important for us to make them understand that the environment is next to them, that it means our life, it means our air, it means everything to us and if anything goes wrong with the environment, then we are affected and we can’t live well, and if we have to live a healthier, longer life, we have to make sure that we are concerned about our environment, and so because we’re able to say that’s true, we were now able to now tie in the issue of second hand smoke to say, okay, even if you clean your whole house, you wash your clothes, you do everything, you sweep, and yet the air that you breathe in is already polluted, it’s also doing lots of damage, … So we were able to now release that to them, and then the fact that we were able to make that connection was a powerful point.

The youth…they were able to make correlations and talk about what in the environment is impacted by [the Creek], wildlife that’s no longer there, were talking about birds, we’re just talking about the things that you would ordinarily see and a clean stream of water that’s no longer there.

### Expansion of Network and Partnerships

All but two grantees talked about how the community grant expanded their network in some way. Community grant funding allowed them to pilot a new program that could potentially be expanded to additional sites (e.g., STEM education in additional schools, a garden program in additional housing authorities), or to add new program components with new partners (e.g., addition of a food co-op program with local community center and organization, or cooking classes with community farmer’s market staff). Funding also provided increased opportunities for volunteer involvement, leading to a new or sustained volunteer base; and increased awareness of the organization’s work (both among grantees and others in the field), which sometimes led to concrete partnerships and additional opportunities. One grantee used funds specifically for community outreach and marketing and as a result established many successful partnerships.

… I have gotten requests to be involved in training other organizations. Some of the churches in the community have reached out to me and said that we want you to work with us and help us work with the young people and our congregation and other organizations because of being at the NPU meeting and touching so many of the organizations. We’ve gotten a lot of response.

For some grantees, it was the funding in and of itself that helped to expand their network:

“…there’s something about just having the mini-grant that has -- it’s really been a catalyst. It’s helped us again with that credibility factor, that Emory and the HERCULES Center would make an investment has kind of turned a lot of heads and raised some eyebrows to the point where people are kind of interested in what it is that we’re doing.”

Grantees also discussed the other ways that partners and partnerships were integral to their grant, such as enhancing supportive interactions. For example, they partnered with a variety of partners to implement their projects, including schools and universities (and their students), a health department, other community organizations, environmental groups, city government, social service organizations, and businesses. These partners provided tangible resources or in-kind services such as participant referrals, volunteers, or project supplies. For example:

And its right next door to [a] Clinic and we’re working with 2 of the doctors there that are going to start referring patients to come and hang out at the garden and learn about … there are a lot of people that are diagnosed with obesity and diabetes and even depression. They start coming to the garden and working and maybe that will help turn their spirits….

we’ve had other people to come and give in kind services, and so I’m learning about these kinds of things. You know, we wanted to go to a Braves game, well, we did, and other people came in and assisted.

### Partnerships also provided opportunities for dissemination, expansion, and leadership related to their programming:

So we have other store partners: Walmart… and then we’re partners with Wayfield Foods that has 9 stores and one of the stores is in our main target area. And both employees will be learning how to help customers and we have “cooking matters” and other kind of store tours to teach people how to shop.

## Conclusions

The HERCULES Community Grant Program showed increased grantee capacity with regards to leadership, sense of community, community power, knowledge and skills, and expanded networks and partnerships. Specifically, the program increased opportunities to take on leadership roles, increased grantee professional skills and visibility as leaders, and increased confidence among grantees. It increased the grantees’ sense of community allowing grantees to build trust, to increase community engaged programming and community awareness of environmental health issues and, in some instances, to connect with youth in the community. Similarly, increased awareness and sense of community combined with opportunities for action led to increased community power defined in this study as increased action or organization around an identified issue. Both grantee organizations and community members increased their knowledge and skills, with grantees specifically gaining skills in grant administration and community members increasing their knowledge of environmental health topics. Lastly, grantees said that the grant gave them the opportunity to build partnerships through program expansion, such as adding new locations or engaging new partners as they added new program components. The grant program also provided the opportunity to expand their volunteer base and increase awareness of their organization. These new partnerships were formed with various types of organizations, many of which also contributed tangible resources.

Community grant programs may be a novel way to support and enhance community capacity. Evaluations and discussion of community capacity rarely include community grant programs, but other health promotion, education, and training interventions (20, 29). The documented increase in community capacity that resulted from this grant program shows that using community capacity to guide programming and evaluation can provide the short-term outcomes that are necessary when tackling long-term environmental health problems. With a focus on responding to environmental threats and improving environmental health at the community level, Freudenberg et al. (20) suggest specific strategies to increase community and organizational capacity, including training and technology transfer, technical assistance (TA), and community organizing/social action, which were all either components of this program or the grantees’ programming. For example, while many grantees described providing training to community members and using community organizing strategies, TA was also an integral component of the grant program itself (25). Future grant programs may want to intentionally incorporate more of these strategies into their programming and TA. In fact, after reviewing the evaluation results presented here and in the paper by Pearson et al. (25) with the SAB, together the HERCULES CEC and SAB have developed a revised community grant program that intentionally incorporates Freudenberg et al.’s strategies of training, technical assistance, community based participatory research (CBPR), and community organizing. This program, the Shaheed DuBois Exposome Roadshow and Community Grant Program (Roadshow and Grant Program), is divided into four strategic phases and funds a group of community residents for three years to organize around an environmental health issue, with training and educational opportunities and technical assistance throughout (30–32).

Designing and assessing programs for community capacity does not come without its complexities. Like Goodman et al. (3), we found conceptual overlap between dimensions and identified multiple relationships between them. For example, grantees described how a sense of community, combined with increased knowledge and awareness of environmental health issues, enhanced the community’s power to act. A “sense of community” is grounded in an understanding of shared values, community cohesiveness, and collective experience (33, 34). With the grant program, an enhanced sense of community allowed members of shared space and place to come together in a sustainable way, represented a catalyst for community change, as well as encouraged collective action in response to environmental stressors. Further, research has indicated that sense of community is positioned as a characteristic of empowerment and as individuals have greater feelings of support and belonging, they are more inclined to take action to address concerns (35). The improved access to resources and support structures that our grantees reported are equally essential for advancing community environmental change: while expanded networks are directly related to an increase in tangible financial capital/resources (which we had already operationalized in our codebook), they also contribute to building leadership capacity by increasing opportunity for support, access to assets, and increased awareness of the organization within the broader community (33–35). Community empowerment literature stresses the importance of participation and gaining control over decisions and resources in addressing political and social change (7).

Despite their conceptual intersections, each individual dimension of capacity that this grant program enhanced shows promise in creating community change and improving community health. To start, this program showed evidence of enhanced leadership among both the grantees and the broader community. Leadership has been shown to be critical to effective community initiatives (36) (37) and may improve and sustain outcomes. For example, both Freudenberg (2) and Minkler (37) and colleagues found leadership to be an essential domain of capacity in policy advocacy and mobilizing action that led to lasting and sustained environmental health improvements. The engagement of youth in such initiatives is particularly impactful, as it not only instills a sense of responsibility and civic duty in young people but also cultivates future leaders poised to sustain and enhance community resilience and social dynamics (38). Furthermore, when leadership is lacking, community initiatives may only have moderate impact and face challenges such as strained relationships, emphasizing the importance of developing leadership skills (39). Many of the grantee projects described here incorporated leadership opportunities for community members by providing volunteer leadership experiences. Future programs may want to further promote leadership development, for example, by offering training or paid employment opportunities that include program co-development and management (40) and incorporating the specific characteristics of effective leadership in community-based initiatives (36). In essence, this kind of community grant program can serve as a pivotal platform for nurturing competent leaders and fostering a resilient, proactive community spirit that sustains results beyond the initial program period.

Our evaluation found that the infusion of grant funding into community projects also bolstered a sense of community, which may also stimulate greater engagement among community members, nurturing trust and forging solid relationships amongst community stakeholders. Similar to the collective impact framework, by addressing shared concerns, community grants can increase awareness of local issues, facilitate creation of a more cohesive community network and collaboration, and incentivize collective involvement (41). This active participation is crucial for devising more efficacious and durable responses to the community identified challenges (42). Grant funding fosters a collaborative environment where communities can work together effectively towards community-defined common goals. In particular, a sense of community may play an integral role in motivating community action and policy change (37). Goodman et al. (3) also acknowledged this interplay between a sense of community and these other domains of community capacity, which they termed “Social and Organizational Networks”, “Community Power”, and “Citizen Participation”, noting that a sense of community fosters trust and cooperation, reinforces social support, and fosters a desire to participate, which reinforces the community’s power base and facilitates collective action.

As noted above, community grants may be powerful tools for empowering members to initiate action, lobby for change, and emerge as leaders within their local domains, assessed in our program as “Community Power”. Through the intentional allocation of resources, community grants can support projects that not only tackle immediate community concerns but also cultivate an environment where individuals feel capable and confident to make a difference in their communities (43, 44) As noted above, community power can be further enhanced by building a sense of community and partnerships, but also by providing education and information that arms residents with the knowledge necessary to confront environmental and community health issues (2, 44). The success of this program in enhancing community power underscores the potential for grants to stimulate social change across varied contexts. Community grants like these may be further improved by adding educational components and linkages to community-engaged research partnerships that enhance networks and provide scientific information, similar to that of our revised Roadshow and Grant Program.

Related to information, this evaluation demonstrated that community grant programs can facilitate knowledge and skills development among grantee organization leadership and community members alike. In particular, the leadership and organizational skills demonstrated by some of our grantees and further developed by the grant programs’ reporting requirements are especially important to project success (2, 3, 36). Regular performance monitoring and the need to achieve certain outcomes enable participants to work towards specific goals, enhancing their problem-solving abilities and confidence (45). Notably, this kind of performance monitoring was integrated into this Community Grant Program by requiring grantees to report progress towards their goals at two timepoints during the grant period. Success in meeting these goals can boost participants’ self-esteem and motivate them to take on larger community roles (46). Notably, other community capacity evaluations haven’t specifically assessed knowledge (2, 27, 36). Our evaluation conceptualized knowledge and skills together, and grantees reported increased knowledge, particularly around environmental health issues. Increased environmental health knowledge and literacy has been linked to behavior change and community change (47) and may be an important component of community grant implementation and evaluation. In fact, our revised community grant program includes components that are specific to increasing organizational skills around planning, organizing, and sustaining efforts as well as increasing knowledge of environmental health through a popular education workshop component.

Lastly, this evaluation demonstrated that community grant programs may foster the expansion of networks, facilitating synergistic partnerships among grantee organizations, community partners, and a range of stakeholders. The horizontal and vertical linkages described by our grantees adds breadth and depth to community mobilization and increases access to resources with potential to influence policy (2). This kind of organic partnership building within communities was identified by Crisp and colleagues (48) as a specific approach to capacity building with potential for positive outcomes. However, they note that the sustainability of these partnerships once funding ends is less known, and as such continued evaluation of partnership, and capacity building approaches in general, is needed.

By increasing community capacity, community grant programs have the potential to enhance community and environmental health. As Minkler and colleagues (37) showed, increased capacity can catalyze community engagement and result in improved environmental health practices, regulations, and policies. Furthermore, environmental health burdens are often interconnected with social determinants of health such as income and neighborhood, underscoring the necessity for policy and institutional changes that require community action (20). The capacity gains documented by this evaluation occurred across a range of projects and settings, indicating that community grant programs may be a scalable strategy for advancing public health that can be replicated in other communities.

### Limitations

While this grant program shows promise for the positive sustained community change described above, it was not without its limitations. Of course, grant programs cannot solve all problems and have their own inherent issues, such as interpersonal dynamics among community members and the limited and short-term funding. We have attempted to address many of these limitations with our new Roadshow and Grant Program, which we will also describe here in conjunction with the specific limitations of this grant program and its evaluation.

While this paper describes the many ways in which this program built or enhanced community capacity, the grantee organizations or their staff were not always members of the community where they implemented their program or project, so the capacity built by participating in this program did not always remain in the community. However, as demonstrated by the evaluation results presented here, there were many examples of increased capacity among participating community members, not just among program staff. Relatedly, while our goal was to prioritize organizations with lower capacity, we did not always succeed in this because groups with more experience may have been better equipped to write a successful grant application. We tried to address this by using a simple, short application, offering a grant writing webinar, and providing pre-review and feedback on applications with the opportunity to revise and re-submit. We also provided detailed feedback to the applicants that were not funded, yet we do not know if their participation in this application process improved their success in future funding opportunities. To prioritize building capacity directly into the community, our new Roadshow and Grant Program funds community groups directly, mobilizing groups of community members to take the lead and identify and address their selected priority issues.

Increased community capacity can improve community protection from environmental hazards (20, 49), yet this program only lasted one year, and this evaluation did not follow these organizations or the participating community members beyond their participation, so we cannot report on the longer-term outcomes. By maintaining contact with past grantees, we are aware of some accomplishments that were not recorded in our systematic evaluation. The small funding amount ($2500) could also be seen as a limitation, yet we believe that this evaluation and results reported previously (25) show how much groups were able to accomplish with this amount; and by limiting the amount we were able to fund more groups, which our SAB prioritized. However, our new program tries to address both limitations, providing community groups with $6000 over three years. The final evaluation point in the new program is at year three, allowing us to assess some longer-term accomplishments, including the development of community-academic research partnerships resulting in further opportunities for improved environmental health.

Other limitations of this evaluation include the possibility of desirability bias because grantees were being interviewed by their funder, and we only interviewed one representative of the grantee organization, whose perspective may have varied from others in the organization or the participating community members. To avoid bias, staff who did not work with the grantees conducted the interviews and transcripts were de-identified. In the evaluation of the new program, we interview two community leads (“liaisons”) and have a focus group with participating community members in order to collect more perspectives.

Lastly, we did not assess all dimensions of community capacity in this evaluation but instead focused on those deemed most relevant by the SAB to our goals for the program, which are also the most commonly used dimensions in other community capacity evaluations (27). As noted above, “Citizen Participation” may be an important aspect of capacity to include in grant program implementation and evaluation, and it has been incorporated into our new Roadshow and Grant Program by encouraging broad participation from a representative group of community members (32) and assessing that participation through document review and interviews and multiple time points. Likewise, Liberato and colleagues (29) found that “Participatory decision-making”, which includes participation of a variety of perspectives, was common and important domain of community capacity. Community capacity reviews (27, 29) may provide further insight into additional dimensions of community capacity that were not considered when designing this program and its evaluation and may serve to guide future community-based programming and evaluations. They may also help elucidate the conceptual overlap between the dimensions of community capacity which we did not systematically analyze in this evaluation yet discovered through inductive analysis.

In conclusion, despite its limitations, the findings from this qualitative analysis shed light on the multifaceted impacts of the HERCULES Community Grant Program on building community capacity within small community-based organizations. The program not only provided financial support but also served as a catalyst for fostering leadership, knowledge, and skills, enhancing a sense of community and community engagement, empowering individuals and communities to act, and expanding networks and partnerships. Through a combination of education and action, grantee organizations and community members alike gained valuable knowledge, skills, and confidence to address pressing issues related to healthy food access, water pollution, and waste disposal. Furthermore, the ripple effects of these initiatives extend beyond the grant period, with the potential to catalyze broader community-level changes and promote sustainable solutions. Overall, the success of this small Community Grant Program underscores the importance of investing in grassroots initiatives and collaborative efforts to enhance community capacity to address complex social and environmental challenges (i.e., the exposome).

## Supplementary Material

Supplementary Files

This is a list of supplementary files associated with this preprint. Click to download.

• AdditionalFile1.InterviewGuide.pdf

Additional files

Additional File 1. Interview Guide.pdf. Interview Guide: the interview guide used to conduct evaluation interviews with grantees of the Community Grant Program.

## Figures and Tables

**Table 1:** Operationalizing the Dimensions of Community Capacity

Community Capacity Dimension	Community Capacity Definition (adapted from Goodman et al. (3) and Freudenberg (2))	Example Interview Guide Question	Operationalized Codebook Definition
Leadership	• Presence of experienced, skilled leaders willing to address environmental health issues • Shaping and cultivating the development of new leaders	• In what ways has the grant provided you with leadership opportunities? • What about for others in the community? • *[If unclear what leadership refers to:] This may include giving presentations, increased visibility for your organization, among other things.*	**Leadership Development** Used when participant discussed how the mini-grant process fostered the development of leadership opportunities for the grantee or community members.
Sense of Community	• High level of concern for community issues • Extent to which participants share an identity related to community as a physical and social environment and a willingness to take action based on that identity	• As a result of the grant program have you built a stronger connection with your community? If so, please explain. • *Probe: Has interest in or awareness of your organization increased? Has membership increased? Is there a greater participation from the neighborhood residents or larger community?*	**Sense of Community & Community Engagement** Used when participant discussed emotional connection with community, engagement in community issues, service to others, community involvement, benefits to the community as a result of the grant.
Community Power	• The ability to create or resist change regarding community turf, interests, or experiences	• In what ways, if any, has the grant strengthened the community's ability to address environmental health concerns?	**Community Power** Used when participant provided examples of how the grant funding increased community awareness, influence and organization around community issues/concerns and/or incited a response or action from the community.
Skills	Level of relevant organizational, scientific, political, and information seeking skills among range of participants	• What are the top 2–3 things you learned as a result of *conducting your project*? • What are the top 2–3 things you learned as a result of *administering the grant*?	**Knowledge and Skills** Used when participant discussed knowledge and skills gained as a result of conducting the project and administering the mini-grant, inclusive of personal benefits and those related to staff, volunteers and partners.
Social and interorganizational networks	Horizontal and vertical linkages among participants and their organizations and other relevant local, regional, and national groups	• As a result of the grant program, has your network expanded at all? [If yes] Tell me about that. • Probe: Has the mini-grant program been helpful in the creation of new partnerships with other organizations, agencies, or programs? If so, please explain.	**Expansion of Network** Used when participant discussed how the mini-grant helped them create new organizational partnerships **Other Partnerships** Used when participants described other ways in which the mini-grant involved partnerships or other community relationships.
Resources	Financial, human, and social resources available for addressing environmental health concerns		

**Table 2.** Grantee characteristics at time of grant

Grantee characteristic	# of grantees (N = 12)
***Topic(s) addressed (not exclusive)***	
Water Pollution	3
Food Access and the Built Environment	5
Agriculture Challenges and Solutions	3
Soil Contamination	1
Knowledge of Local Environmental Issues	4
Abandoned Buildings/Sites	1
Waste Disposal/Illegal Dumping	5
**Location in the Metro-Atlanta Area**	
South and Southwest Atlanta	6
North and Northwest Atlanta	2
Dekalb County	2
City of Clarkston	2
**# of staff**	
0–5	7
6–10	4
11 +	1
**Annual Budget** *(n = 10)* ^a^	
$0–$10,000	5
$11,000 – $20,000	1
$21,000 – $50,000	0
$51,000 – $100,000	1
$101,000 – $499,000	2
$500,000+	1
**Existing partnerships**	
0–5	9
6–10	1
11–20	1
20+	1
**Years of exstence as an organization**	
0–5	5
6–10	4
11–20	1
20+	2

a Annual budget was not collected systematically on each year of the application, though some reported it elsewhere.

## Data Availability

The datasets used and/or analysed during the current study are available from the corresponding author on reasonable request.

## Abbreviations

CBPR: community based participatory research
CEC: Community Engagement Core
Community Grant Program: “Shaheed” DuBois Community Grant Program
HERCULES: HERCULES Exposome Research Center
SAB: Stakeholder Advisory Board
TTAC: The Tobacco Technical Assistance Consortium
